# Criteria Used in Clinical Practice to Guide Immunosuppressive Treatment in Patients with Primary Sclerosing Cholangitis

**DOI:** 10.1371/journal.pone.0140525

**Published:** 2015-10-21

**Authors:** Kornelius Schulze, Tobias J. Weismüller, Michael Bubenheim, Peter Huebener, Roman Zenouzi, Henrike Lenzen, Christian Rupp, Daniel Gotthardt, Philipp de Leuw, Andreas Teufel, Vincent Zimmer, Florian P. Reiter, Christian Rust, Lars Tharun, Alexander Quaas, Sören A. Weidemann, Frank Lammert, Christoph Sarrazin, Michael P. Manns, Ansgar W. Lohse, Christoph Schramm, German PSC Study Group

**Affiliations:** 1 I. Department of Medicine, University Medical Center Hamburg-Eppendorf, Hamburg, Germany; 2 Department of Internal Medicine I, University of Bonn, Bonn, Germany; 3 Clinic of Gastroenterology, Hepatology, and Endocrinology, Hannover Medical School, Hannover, Germany; 4 Department of Biostatistics, Rouen University Hospital-Charles Nicolle, Rouen, France; 5 IV. Department of Medicine, University Medical Center Heidelberg, Heidelberg, Germany; 6 I. Department of Medicine, University Medical Center Frankfurt, Frankfurt am Main, Germany; 7 I. Department of Medicine, University Medical Center Mainz, Mainz, Germany; 8 Department of Medicine, University Hospital Regensburg, Regensburg, Germany; 9 II. Department of Medicine, Saarland University Medical Center Homburg, Homburg, Germany; 10 Department of Medicine II, Liver Center Munich, University of Munich, Grosshadern Campus, Munich, Germany; 11 Department of Pathology, University Medical Center Hamburg-Eppendorf, Hamburg, Germany; 12 Department of Pathology, University Medical Center Cologne, Cologne, Germany; Texas A&M Health Science Center, UNITED STATES

## Abstract

**Background & Aims:**

Current guidelines recommend immunosuppressive treatment (IT) in patients with primary sclerosing cholangitis (PSC) and elevated aminotransferase levels more than five times the upper limit of normal and elevated serum IgG-levels above twice the upper limit of normal. Since there is no evidence to support this recommendation, we aimed to assess the criteria that guided clinicians in clinical practice to initiate IT in patients with previously diagnosed PSC.

**Methods:**

This is a retrospective analysis of 196 PSC patients from seven German hepatology centers, of whom 36 patients had received IT solely for their liver disease during the course of PSC. Analyses were carried out using methods for competing risks.

**Results:**

A simplified autoimmune hepatitis (AIH) score >5 (HR of 36, p<0.0001) and a modified histological activity index (mHAI) greater than 3/18 points (HR 3.6, p = 0.0274) were associated with the initiation of IT during the course of PSC. Of note, PSC patients who subsequently received IT differed already at the time of PSC diagnosis from those patients, who did not receive IT during follow-up: they presented with increased levels of IgG (p = 0.004) and more frequently had clinical signs of cirrhosis (p = 0.0002).

**Conclusions:**

This is the first study which investigates the parameters associated with IT in patients with PSC in clinical practice. A simplified AIH score >5 and a mHAI score >3, suggesting concomitant features of AIH, influenced the decision to introduce IT during the course of PSC. In German clinical practice, the cutoffs used to guide IT may be lower than recommended by current guidelines.

## Introduction

Primary sclerosing cholangitis (PSC) is a chronic and progressive inflammatory disease of the intra- and/or extrahepatic bile ducts leading to end stage liver disease, transplantation or death within 10–20 years. PSC is associated with dysregulated immune reactions, and activated T-cells can be found in the livers of these patients.[1, 2] This immune imbalance may be a general feature of PSC; however a subgroup of patients can present with or will develop typical features of additional autoimmune hepatitis (AIH) during the course of their disease [also termed overlap syndrome (OS)]. Whereas most experts agree that PSC patients with features of AIH could benefit from immunosuppressive treatment (IT), no consensus has been reached on how to identify these rare patients. The decision to introduce IT in PSC is especially difficult in patients who present with classical PSC at first sight and develop features of increased liver inflammation during the course of their disease. An elevation of serum aminotransferase levels above 5x the upper limit of normal (ULN) and an elevation of serum IgG levels above 2x the ULN have been proposed as diagnostic features pointing towards additional AIH, which should then lead to additional IT.[3] However, in clinical practice most physicians tend to initiate IT at lower serum levels of aminotransferases and IgG.

Liver histology is mandatory to diagnose AIH in patients without PSC, but it is unknown which grade of liver inflammation in PSC indicates additional AIH or an inflammatory activity of PSC which should prompt additional IT.[4] Moreover, given the hypothesis that suppression of liver inflammation could reduce disease progression, it may be important to identify patients at high risk of an inflammatory course of the disease already at diagnosis.

Patients who present with or will develop typical features of AIH during the course of their disease represent rare variants and data on the management of these patients is scarce. Moreover, to date a generally accepted definition of features justifying the additional diagnosis of AIH or indicating liver inflammation that exceeds the degree which is normally seen in PSC is lacking. Since prospective trials will be very difficult to perform in this group of patients we–in a first step–aimed to investigate parameters associated with the introduction of IT in real life treatment in several German hepatology centers. By the identification of factors guiding clinicians in practice to introduce IT in PSC, it may be possible to identify a subpopulation of patients qualifying for future clinical trials on immunomodulatory treatment.

## Patients and Methods

### Patients

Since the aim of this study was to identify parameters associated with the initiation of IT in patients with previously diagnosed PSC in clinical practice, records of 218 patients with established PSC were reviewed between the years 1986 and 2012 at seven university medical centers in Germany. The diagnosis of PSC was established according to accepted criteria using endoscopic retrograde cholangiography (ERC) and/or magnetic resonance cholangio-pancreaticography (MRCP) as described in the European association for the study of the liver (EASL) clinical practice guidelines.[5] Patients with the small duct variant were not included leading to the exclusion of 2 patients due to a normal cholangiogram. Although it is current standard in Germany to treat all PSC patients with ursodeoxycholic acid (UDCA) at a dose of 15–20 mg/kg body weight, 3 patients had to be excluded because of lacking information on UDCA treatment after PSC diagnosis and 8 patients did not receive UDCA during follow-up. Since patients with a simultaneous diagnosis of PSC and AIH were not included into this study, another 3 patients had to be excluded.

Six additional patients, who had a follow-up of less than 12 weeks after the diagnosis of PSC, were excluded. To reach our principal aim, the retrospective cohort is hence formed by 196 PSC patients.

In all patients, the prescription of UDCA is considered to coincide with the time point of diagnosis and this is the starting point for our subsequent analyses. Laboratory and serological parameters were assessed at the start of UDCA treatment and before the start of IS treatment (steroids and/or azathioprine) by standard methods. Among the 196 patients in our cohort, 36 patients were identified who received IT specifically directed towards their liver disease at least 12 weeks after the diagnosis of PSC were identified. These patients will be termed PSC-IT for the purpose of this manuscript.

The presence or absence of inflammatory bowel disease (IBD) was assessed using colonoscopy, and signs of liver cirrhosis (ascites, splenomegaly, and/or esophageal varices) were recorded. Viral hepatitis was excluded serologically. The simplified AIH score, a diagnostic tool to measure the probability of underlying AIH (≥6 points probable, ≥7 points definite AIH) in patients with liver disease, was calculated considering following parameters: ANA or SMA ≥1:40 1 point; ANA or SMA ≥1:80 or LKM ≥1:40 or SLA positive 2 points; IgG >upper normal limit 1 point; IgG >1.10 times upper normal limit 2 points; liver histology compatible with AIH 1 point; liver histology typical for AIH 2 points; absence of viral hepatitis 1 point.[6] Since patient acquisition dates back many years, serum IgG4-levels were not available for analysis.

### Liver histology

Liver tissue of 36 classical PSC patients and 16 PSC-IT patients was available for re-analysis by an expert liver pathologist who was blinded to patient's treatment status. These samples were obtained at diagnosis of PSC and before the start of IT, and examined for the presence of features of AIH as well as PSC. Histological activity was graded according to the modified hepatic activity index (mHAI) and staged according to the criteria of Ludwig.[7, 8] The following histological features were investigated to indicate PSC: bile duct damage, bile duct basement membrane thickening, periductal concentric fibrosis, ductopenia / fibro-obliterative scars, ductular reaction, periportal copper deposition, and metaplastic CK7-positivity of periportal hepatocytes. The following histological features were investigated to indicate AIH: portal inflammation, lymphoplasmocytic infiltrate, interface activity, lobular inflammation, rosette formation, and emperipolesis.[6]

### Statistical analysis

The general aim of the present cohort study was to investigate whether clinical, biochemical, or histological data were associated with the initiation of IT during the PSC disease course.

Patient's time under risk for the initiation of IT started at first UDCA prescription and ended at start of IT, liver transplantation, death, or end of follow-up. Since starting the IT implies survival and no transplantation beforehand, analyses were carried out using methods for competing risks.[9] Hence, figures on the initiation of IT indicate the cumulative probability receiving IT in the present of competing risks considering relevant cut-off values. Patient’s characteristics of at least ordinal level were described using the median accompanied by the first (Q1) and the third quartile (Q3).

As this study is exploratory by nature, no correction for multiple testing was carried out, and a p-value less than 0.05 was considered to be statistically significant. Underlying raw data is included in the supporting information (S1 Table). The study protocol was approved by the Ethics Committee of the Hamburg Medical Association and the patient records were anonymized and de-identified prior to analysis.

## Results

### Patient population

The study population consisted of 196 PSC patients of whom 36 received IT. The median follow-up period until liver transplantation, death or censoring of the 196 patients was 3.5 years (Q1: 1.7 years, Q3: 7.6 years). In PSC patients who received IT during the course of their disease, IT was started a median time of 2.7 years (Q1: 1.6 years, Q3: 6.0 years) after PSC diagnosis.

### Patient characteristics at first presentation

The median age at PSC diagnosis was 34 years and 65% of patients were male. Although one might expect that younger patients present with a more inflammatory course of PSC, in this analysis age at diagnosis (29 years) did not appear to be associated with subsequent IT. Gender was also not associated with IT (p = 0.77). IBD was present in 48% of all 196 patients at PSC diagnosis, but after screening by colonoscopy, 59% of PSC-IT patients and 67% of all other patients had an IBD-diagnosis. The presence of IBD during follow-up was not significantly associated with IT (p = 0.68). And finally, the ratio between ALT and AP >2.5 with respect to normal values was not significantly different in patients receiving IT in the course of the disease. Table 1 summarizes the clinical characteristics of the cohort.

**Table 1:** Univariate analysis of potential risk factors for IT as first event after UDCA prescription (using Cox' model) at first presentation.

State at the end of follow-up	PSC-IT	PSC		95% Confidence Interval		p-Value
			Hazard Ratio	Lower Limit	Upper Limit	
**All patients [N]**	36	160				
**Median age [Years]:**	35	35				
**Age groups [N, (%)]:**						
[4–25 years)	13 (36.1)	29 (18.1)	1.7	0.8	3.6	0.19
[25–40 years)	13 (36.1)	72 (45.0)				
[40–72 years]	10 (27.8)	59 (36.9)	1.2	0.5	2.8	0.63
**Sex [N (%)]:**						
male	24 (66.7)	104 (63.8)	0.9	0.5	1.8	0.77
female	12 (33.3)	56 (36.2)				
**IBD [N (%)]**	**20** * **(58.8)**	**100** * **(66.7)**	0.9	0.4	1.7	0.68
Ulcerative colitis	18 (90.0)	68 (68.2)				
Crohn’s disease	2 (10.0)	18 (18.0)				
Indeterminate colitis	-	14 (14.0)				
**ALT vs. AP Ratio >2.5 times [N (%)]:**	-	6 (4.0)	0.0	0.0	.	0.99
**Clinical signs of cirrhosis [N (%)]:**	23 (69.7)	37 (29.4)	4.1	2.0	8.7	< 0.01

* = after first screening by colonoscopy

### Parameters associated with immunosuppressive treatment in patients with PSC

#### Histological hepatitis activity

**Fig 1:**
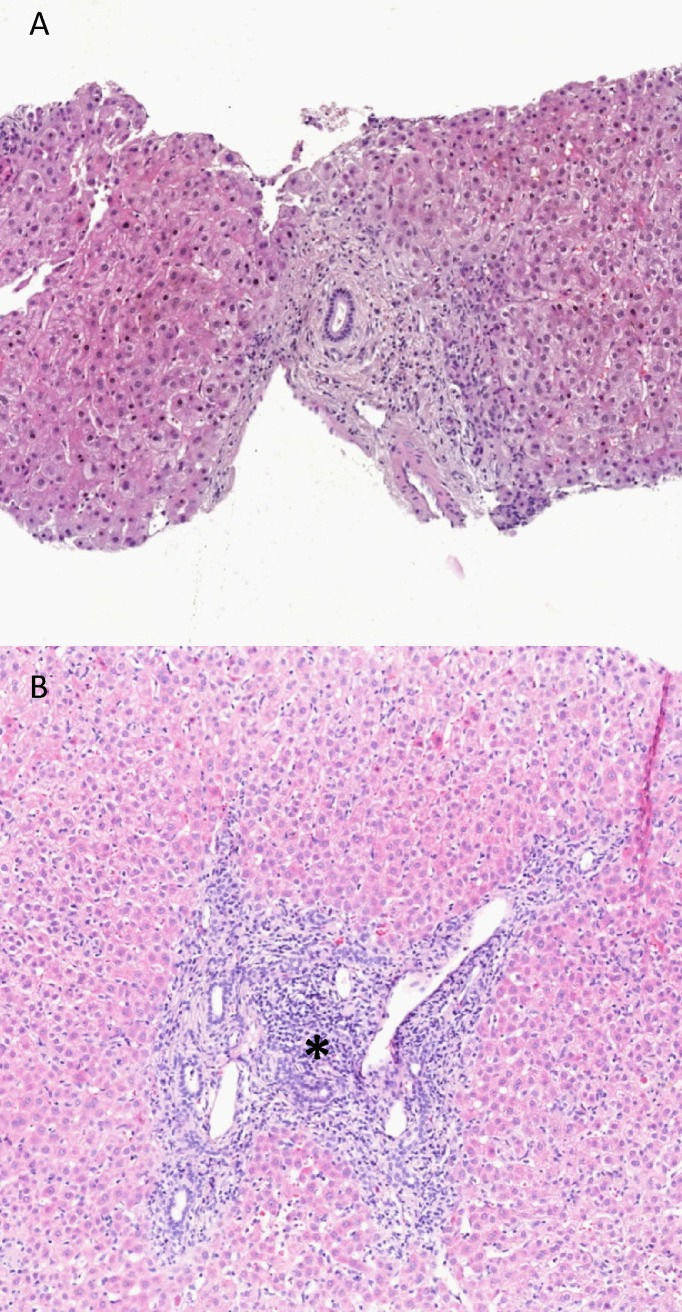
Liver histologies of PSC patients with a mHAI score greater and lower than 3 points. A: typical concentric fibrosis and low grade of inflammation. B: typical periductular fibrosis and additionally signs of interface hepatitis (*), representing a patient with a mHAI score of 8/18. Staining: hematoxylin eosin.

Histological findings are key to the diagnosis of classical AIH and histological disease activity scores are used by many experts to guide IT. We therefore assessed the severity of hepatitis as measured by the mHAI. For this purpose we were able to reassess liver biopsies obtained at diagnosis of PSC for a subset of 52 out of the 196 patients. Among these patients, 16 received IT. Table 2 summarizes the histological findings in both groups of patients. Twelve out of 52 histological samples demonstrated mHAI scores of more than 3/18 points. Eleven of these 12 cases subsequently received IT during the course of disease. Of the remaining 40 cases (mHAI less or equal than 3 points), only 5 patients were treated with IT during the observation period. Thus, taking the mHAI score as a probability factor into account, the cut-off value of only 3 points was significantly associated with the introduction of IT during the disease course of PSC [hazard ratio (HR) 3.6, p = 0.0274, Table 3]. Fig 1 highlights two typical liver histologies of patients with a mHAI score greater and lower than 3 points.

**Table 2:** Histological characteristics in patients with and without IT.

	PSC-IT [n = 16]		PSC [n = 36]	
	Patients [N (%)]	Median (Q1, Q3)	Patients [N (%)]	Median (Q1, Q3)
**Patients:**	**16 (31)**		**36 (69)**	
**Fibrosis [Grade 1–6]:**	** **	**4 (2, 5)**		**2 (1, 3)**
**Concentric fibrosis:**	**7 (44)**		**21 (58)**	
**mHAI Score** * **[0–18]:**		**4 (3, 6)**		**1 (1, 2)**
A [0–4]		1 (1, 1)		0 (0, 0)
B [0–6]		0 (0, 0)		0 (0, 0)
C [0–4]		1 (1, 2)		0 (0, 1)
D [0–4]		2 (2, 2)		1 (1, 1)
**Histological diagnosis of PSC/AIH overlap:**				
No	4 (25)		35 (97)	
Probable	7 (44)		1 (3)	
Typical	5 (31)		0 (0)	
**Presence of pseudorosettes:**	12 (75)		24 (67)	

* *A*: *periportal or periseptal interface hepatitis (piecemeal necrosis); B*: *confluent necrosis; C*: *focal lytic necrosis*, *apoptosis*, *and focal inflammation; D*: *portal inflammation*.

**Table 3:** Histological disease activity and simplified AIH score as single risk factors for IT in patients with PSC.

	Complete Cohort	PSC-IT Cohort		95% Confidence Interval		*p-Value*
	Patients [N (%)]	Patients [N (%)]	Hazard Ratio	Lower Limit	Upper Limit	Cox
**mHAI Score**	**52 (100)**	**16 (100)**				**0.03**
≤3 points	40 (77)	5 (31)				
>3 points	12 (23)	11 (69)	3.6	1.2	11.1	
**Simplified AIH Score** *	**122 (100)**	**17 (100)**				**<0.0001**
≤ 5 points	100 (82)	2 (11)				
> 5 points	22 (18)	15 (89)	36.4	8.3	159.7	

* Including patients with/without histological assessment considering maximum points of histological score still leading to ≤ 5 points.

#### Simplified AIH score

The simplified AIH score can be used in clinical practice to aid the diagnosis of classical AIH.(6) Hence, we evaluated whether this score was associated with subsequent IT in patients with PSC. Using a cut-off value of more than 5 points–determining a probable or definite AIH diagnosis in patients with chronic hepatitis–the simplified AIH score was demonstrating the strongest association to the subsequent initiation of IT in patients with PSC (HR 36, p<0.0001, Table 3). In total, 68% of patients with a simplified AIH score of more than 5 points received IT for their liver disease during the course of the observation period.

### Parameters at the time of PSC diagnosis which were associated with subsequent immunosuppressive treatment

#### Laboratory values and presence of cirrhosis at the time of PSC diagnosis

Next, we were interested whether laboratory values already at the time of PSC diagnosis were associated with the subsequent introduction of IT in patients with PSC. To this end, biochemical markers of cholestasis as well as hepatitis activity were analyzed (Table 4).

**Table 4:** Laboratory values at the time of PSC diagnosis and the association with the subsequent introduction of IT during the course of PSC.

	Complete Cohort	PSC-IT		95% Confidence Interval		*p-Value*
	Patients* [N* (%)]	Patients* [N* %)]	Hazard Ratio	Lower Limit	Upper Limit	
**AP, Cut-off 200 U/L**	**147 (100)**	**18 (100)**				
≤	**53 (36)**	**3 (17)**				
>	**94 (64)**	**15 (83)**	**2.0**	0.6	7.0	0.29
**yGT, Cut-off 200 U/L**	**152 (100)**	**20 (100)**				
≤	**69 (45)**	**6 (30)**				
>	**83 (55)**	**14 (70)**	**2.4**	0.9	6.3	0.07
**Bilirubin, Cut-off 1.2 mg/dL**	**144 (100)**	**19 (100)**				
≤	**94 (65)**	**7 (37)**				
>	**50 (35)**	**12 (63)**	**2.6**	1.0	6.6	0.05
**AST, Cut-off 70 U/L**	**102 (100)**	**12 (100)**				
≤	**75 (74)**	**6 (50)**				
>	**27 (26)**	**6 (50)**	**2.4**	0.8	7.3	0.14
**ALT, Cut-off 70 U/L**	**149 (100)**	**20 (100)**				
≤	**71 (48)**	**5 (25)**				
>	**78 (52)**	**15 (75)**	**2.7**	1.0	7.5	0.05
**IgG, Cut-off 16 g/L**	**101(100)**	**15 (100)**				
≤	**66 (65)**	**4 (27)**				
>	**35 (35)**	**11 (73)**	**5.3**	1.7	16.5	**<0.01**
**At least one positive Auto-AB-titer**	**82 (100)**	**8 (100)**				
–	**33 (40)**	**2 (25)**				
+	**49 (60)**	**6 (75)**	**3.6**	0.7	18.4	0.13
**Creatinine, Cut-off 0.8 mg/dL**	**136 (100)**	**18 (100)**				
≤	**90 (66)**	**10 (56)**				
>	**46 (34)**	**8 (44)**	**2.7**	1.1	7.1	**0.04**

* Patients with values available at the time of PSC diagnosis

**Fig 2:**
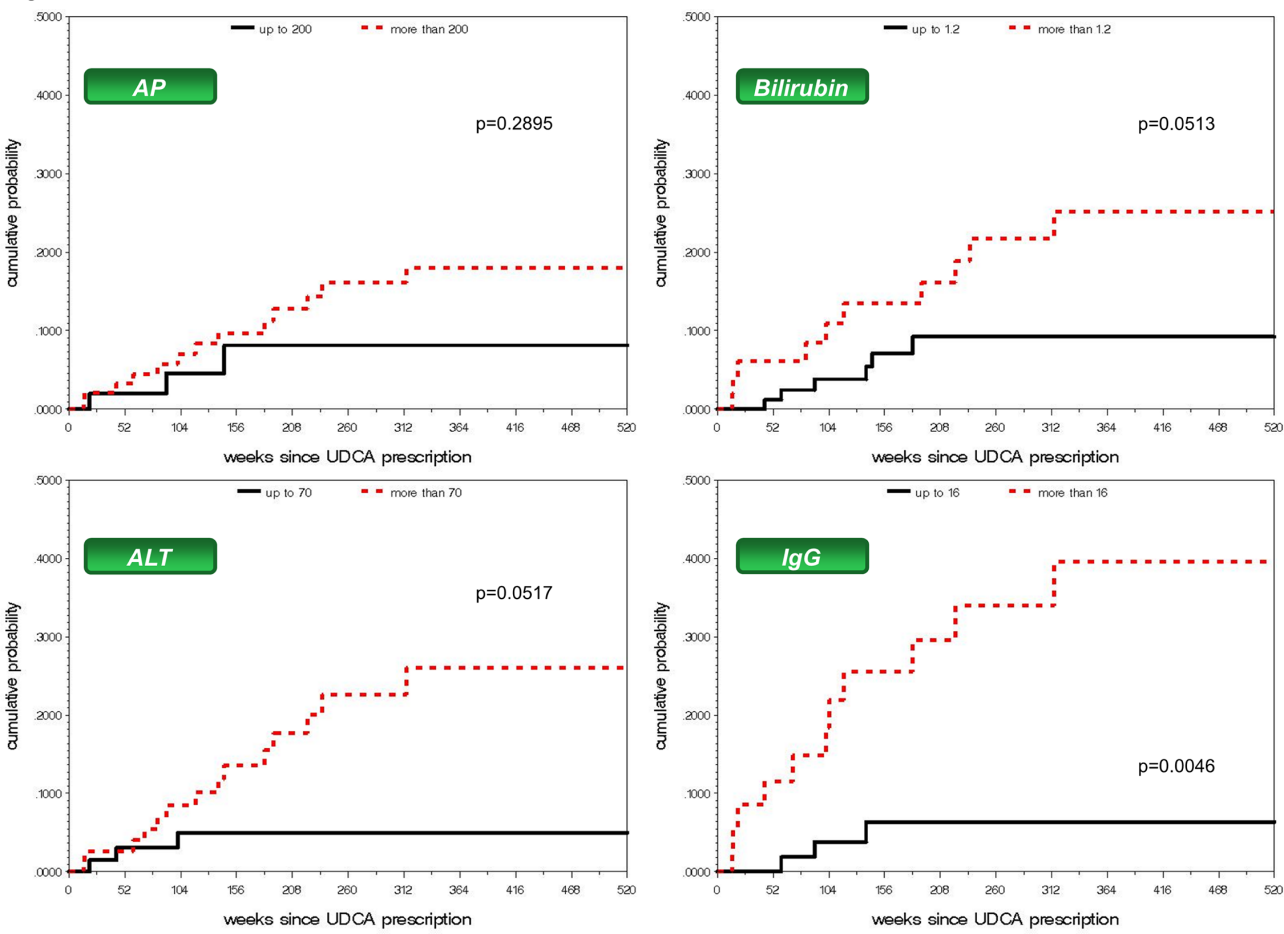
Cumulative probability of introduction of IT in the presence of competing risks at the time of PSC diagnosis (start of UDCA prescription): Alkaline phosphatase (AP), bilirubin, alanine aminotransferase (ALT), immunoglobulin G (IgG).

In patients with established AIH, serum IgG levels were described to correlate with histological disease activity.[10] In our PSC cohort, using a cut-off value of 16 g/L ULN, IgG at the time of initial presentation was associated with IT during the course of disease: among patients with IgG levels above 16 g/L at PSC diagnosis, 31% received this treatment during follow-up (HR 5.3, p = 0.0046, Fig 2), suggesting an increased hepatitis activity in these patients already at the time of PSC diagnosis.

Patients with higher hepatic inflammatory activity may more rapidly develop liver fibrosis and present a higher rate of liver cirrhosis at PSC diagnosis. Indeed, signs of liver cirrhosis at PSC diagnosis were associated with an increased probability of subsequent IT (HR = 4.1, p = 0.0002).

Serum bilirubin levels above 1.2 mg/dL (ULN, Fig 2) as well as an ALT value of more than 70 U/L (2x ULN) tended to be associated with a higher probability for the introduction of IT during the course of disease (p = 0.05 each, Table 4). The frequency of autoantibody titers was not significantly higher in patients with IT, but they were infrequently available at the time of PSC diagnosis (p = 0.128). Markers of cholestasis, such as levels of serum alkaline phosphatase or γ-glutamyltransferase, were not found to be associated with subsequent IT. Fig 2 displays the cumulative probability receiving IT during the course of PSC depending on laboratory findings at the time of PSC diagnosis.

## Discussion

PSC can present with varying degrees of liver inflammation, in few patients fulfilling diagnostic criteria of classical AIH. To date, it remains undetermined, which degree of liver inflammation is acceptable for the sole diagnosis of PSC and what defines the need for additional IT. In current guidelines, it is recommended that a cut-off of ALT levels more than five times the ULN and IgG levels above twice the ULN should lead to IT.[3] We here aimed to assess parameters, which prompted the addition of IT during the course of PSC in real life, since we had the impression that in clinical practice, IT is initiated at lower cut-offs than given in guidelines. Although this study was not designed to show whether IT was beneficial for the patients who had received it, the results may help to define patients with PSC who could be included in clinical trials on immunomodulatory treatment.

We here demonstrate that serum IgG levels and the frequency of liver cirrhosis differed already at the time of PSC diagnosis in patients who later received IT. Assuming that early suppression of liver inflammation may slow the progression of the disease; these findings may have practical implications for the early detection of patients at risk. The strongest parameter associated with initiation of IT during the course of PSC was the simplified AIH score which represents a combination of typical features of AIH, including liver histology. Although this in itself may not be surprising it is important to note that the cut-offs proposed in guidelines with regard to ALT serum levels were not associated with IT in this study.

The study presented here has several strengths and obvious shortcomings. A limitation of the present study is the retrospective cohort compilation reaching back until the 1980s, which led to missing clinical data in some of the patients, including serum IgG4 levels, which should be determined at diagnosis of PSC.[3] Also, by study design, the prevalence of patients treated with IT cannot be calculated from this set of data. Most importantly, as mentioned above, this study was not intended to provide evidence for a beneficial effect of IT in inflammatory type of PSC. To our knowledge, this is the largest study to date investigating parameters leading to liver-directed IT in patients with known PSC. The multicenter design should in part correct for individual physician´s preferences in prescribing this treatment, and the patient collective represents a typical PSC cohort including a typical age at diagnosis, IBD prevalence, as well as male predominance of 65%.[5]

This multicenter experience suggests that clinical practice, at least in Germany, is different to what is suggested in guidelines, taken into consideration that the latest position statement of the International Autoimmune Hepatitis Group (IAIHG) suggested an at least five-fold elevation of aminotransferase levels and a more than two-fold elevation of serum IgG levels as features which should lead to additional IT [3, 5, 11]: In the group of 36 PSC-IT patients described here, the median serum IgG level was 23 g/l and the median ALT level was 111 U/l at the time of PSC diagnosis (data not shown).

Scoring systems developed for AIH have been applied to assess the frequency of AIH features in PSC populations.[3, 12–15] These studies demonstrated that features yielding a “definite” AIH diagnosis in patients with PSC patients are rare. Depending on the population analyzed, the original, the revised and, recently also the simplified AIH score identified less than 5% of PSC patients as also harboring typical AIH features.[16] It should be underlined that these scoring systems, especially the original and revised scoring systems, were not developed to differentiate AIH from variant syndromes, such as overlap with PSC or primary biliary cirrhosis, and have not been recommended for this purpose.[3, 17] Nevertheless, in this study it was interesting to see that the simplified AIH score seemed to guide clinicians towards IT, since the hazard to receive IT was 36 times increased for patients with a simplified AIH score of more than 5 points, indicating per definition probable or definite AIH.[6] As a word of caution, liver biopsies could be reviewed in a standardized fashion in 52 patients only and therefore the simplified AIH score could be calculated only for a subset of patients who were not drawn at random from the entire cohort.

Liver histology is an essential part of AIH diagnosis and included in the simplified AIH score.[6] The indication to obtain a liver biopsy is individualized and it is not a routine procedure advised in PSC patients in the current guidelines.[5, 11] However, from the guidelines it remains unclear which PSC patients should undergo liver biopsy in order to assess inflammatory activity. In this study, a mHAI score of more than three points was associated with a three-fold higher risk to subsequently receive IT. Although there is no evidence from controlled clinical trials, it is argued in current guidelines and suggested from small case series that PSC patients with features of AIH could benefit from IT.[3, 5, 11, 18] To identify these patients as early as possible may help to improve their prognosis. The results presented here suggest that elevated serum IgG levels and signs of liver cirrhosis at diagnosis may indicate patients at risk.

## Conclusion

In conclusion, we here describe parameters which were associated with IT during the course of PSC. To date there is no medical treatment with a proven benefit on the course of PSC. The results of the current retrospective cohort study could help to identify patients qualifying for clinical trials targeting liver inflammation. Additionally, since prospective controlled clinical trials are difficult to perform in this rare patient population, the comparison of patients included in multicenter databases with the risk factors identified herein who were or were not treated with IT could indicate an effect of IT on the course of disease.

## Supporting Information

S1 Table Supplementary Information 1_Table_Raw Data. (XLSX)
